# Inflammatory factors and amyloid β-induced microglial polarization promote inflammatory crosstalk with astrocytes

**DOI:** 10.18632/aging.103663

**Published:** 2020-11-16

**Authors:** Lushuang Xie, Ning Zhang, Qun Zhang, Chenyu Li, Aaron F. Sandhu, George Williams III, Sirui Lin, PeiRan Lv, Yi Liu, Qiaofeng Wu, Shuguang Yu

**Affiliations:** 1 College of Basic Medicine, Chengdu University of Traditional Chinese Medicine, Chengdu 610075, Sichuan, China; 2 Institute of Acupuncture and Homeostasis Regulation, Chengdu University of Traditional Chinese Medicine, Chengdu 610075, Sichuan, China; 3 Acupuncture and Moxibustion College, Chengdu University of Traditional Chinese Medicine, Chengdu 610075, Sichuan, China; 4 Neuroscience, College of Arts and Sciences, Boston University, Boston, MA 02215, USA; 5 Department of Neurology, Dalian Municipal Central Hospital, Affiliated Hospital of Dalian Medical University, Dalian 116033, China

**Keywords:** microglia, astrocyte, Alzheimer’s disease, polarization, crosstalk

## Abstract

The immunological responses are a key pathological factor in Alzheimer’s disease (AD). We hypothesized that microglial polarization alters microglia-astrocyte immune interactions in AD. M1 and M2 microglia were isolated from primary rat microglia and were confirmed to secrete pro-inflammatory and anti-inflammatory factors, respectively. Primary rat astrocytes were co-cultured with M1 or M2 microglial medium. M1 microglial medium increased astrocyte production of pro-inflammatory factors (interleukin [IL]-1β, tumor necrosis factor α and IL-6), while M2 microglial medium enhanced astrocyte production of anti-inflammatory factors (IL-4 and IL-10). To analyze the crosstalk between microglia and astrocytes after microglial polarization specifically in AD, we co-cultured astrocytes with medium from microglia treated with amyloid-β (Aβ) alone or in combination with other inflammatory substances. Aβ alone and Aβ combined with lipopolysaccharide/interferon-γ induced pro-inflammatory activity in M1 microglia and astrocytes, whereas IL-4/IL-13 inhibited Aβ-induced pro-inflammatory activity. Nuclear factor κB p65 was upregulated in M1 microglia and pro-inflammatory astrocytes, while Stat6 was upregulated in M2 microglia and anti-inflammatory astrocytes. These results provide direct evidence that microglial polarization governs communication between microglia and astrocytes, and that AD debris alters this crosstalk.

## INTRODUCTION

Glia such as astrocytes, microglia, oligodendrocytes and ependymocytes are significant components of the central nervous system (CNS). These cells sustain neuronal homeostasis, maintain various microenvironments and provide nutrition or energy. The communication between astrocytes and microglia influences many neuronal activities, including neurogenesis and synaptic transmission [1, 2], and regulates messenger molecules such as cytokines, neurotransmitters and metabolites. Factors originating from microglia, including pro-inflammatory proteins, anti-inflammatory proteins and oxidative stress substances, can trigger astrocyte receptors and thus induce inflammation [3]. Microglia can polarize into two subtypes: M1 (via classical activation) and M2 (via alternative activation) [4]. However, it is unclear whether the polarization of microglia impacts their communication with astrocytes.

The activation of microglia and astrocytes is the primary immune response of the CNS, and is involved in the pathological process of Alzheimer’s disease (AD). The reactivity of astrocytes and microglia was observed in APP/PS1 mice (an AD model). When these mice were 16 months old, they displayed abnormal microglia with enlarged cell bodies and shorter, thicker and fewer processes. Moreover, glial fibrillary acidic protein-positive cells (astrocytes) were clustered in the hippocampus [5]. AD products such as amyloid-beta peptide (Aβ) may induce microglial activation to the M1 phenotype. For instance, an *in vitro* study demonstrated that the microglial activity marker CD68 and the M1 phenotype markers tumor necrosis factor alpha (TNF-α), interleukin (IL)-1β and CD86 were upregulated in Aβ-stimulated primary microglia [6]. In another study, the levels of M1 microglia increased after Aβ was injected into the hippocampus in mice [7]. Additionally, Tau was found to induce complement 3 (C3) secretion through the nuclear factor (NF)-κB pathway in astrocytes. Astrocytic C3 was shown to combine with microglial C3 receptors, thus inhibiting debris removal and inducing Aβ and Tau accumulation [8, 9]. However, it is not known whether AD debris alters the communication between astrocytes and microglia during microglial polarization.

Toll-like receptor 4 (TLR4) is a critical participant in microglial activation and polarization [10]. NF-κB signal transducer and activator of transcription 6 (Stat6) are downstream regulatory molecules of TLR4, and are also involved in glial inflammation [10, 11]. While the TLR4/NF-κB pathway polarizes M1 microglia and promotes inflammatory cytokine secretion [11], Stat6 induces microglia toward a beneficial phenotype and improves phagocyte clearance as a downstream factor of IL-4 [12]. The balance between Stat6 and Stat1 seems to determine the microglial phenotype, as autophagy inhibitors promote anti-inflammatory microglial polarization by upregulating Stat6 and downregulating Stat1 [10]. The NF-κB and Stat6 pathways are also activated during astrocyte inflammation. Excretory/secretory products activate the NF-κB pathway, which causes astrocytes to release IL-1β and IL-6 [13]. On the other hand, through the Stat6 pathway, astrocytes of the A2 phenotype produce antioxidant factors such as nuclear factor erythroid 2 and arginase-1 (Arg1) [14].

In this study, we used different inflammatory stimuli to promote M1/M2 microglial polarization, and studied the interactions of astrocytes with the polarized microglia. Furthermore, we used Aβ as simulate AD debris, and examined its effects on microglial polarization and microglia-astrocyte interactions. We measured NF-κB and Stat6 levels in microglia and astrocytes to examine the mechanism of inflammation.

## RESULTS

### Inflammatory factors activated microglia and directed their polarization

**Figure 1:**
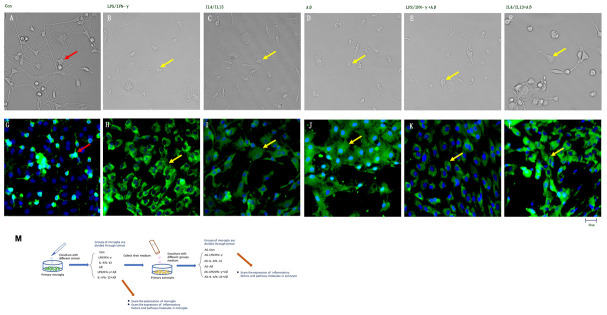
**Inflammatory factors and Aβ activated primary microglia *in vitro*.** (**A**–**F**) Microglial morphology was observed in light field images. (**G**–**L**) Microglia treated with different stimuli were stained with an anti-Iba1 antibody and observed on a fluorescence microscope. Green represents Iba1^+^ cells, while blue represents DAPI staining. (**A**, **G**) Control microglia. (**B**, **H**) Microglia treated with LPS/IFN-γ for 24 h. (**C**, **I**) Microglia treated with IL-4/IL-13 for 24 h. (**D**, **J**) Microglia treated with Aβ1-24 for 24 h. (**E**, **K**) Microglia treated with Aβ1-24 for 2 h, and then with LPS/IFN-γ for 24 h. (**F**, **L**) Microglia treated with Aβ1-24 for 2 h, and then with IL-4/IL-13 for 2 h. Scale bars, 25 μm. (**M**) Description of methods: Primary microglia were divided into six groups to be treated with different stimuli. Media from these microglia were then collected and co-cultured with primary astrocytes, and these astrocytes were divided into six groups according to the media with which they were treated. Microglial polarization was examined, and cytokine levels in both types of glia were measured.

We obtained primary glial cells from newborn rats, and isolated primary microglia from the mixed cell cultures. The primary microglia were sometimes rounded and sometimes ovular in shape, and had spindle process. Then, we stimulated the microglia with lipopolysaccharide (LPS) and interferon (IFN)-γ to polarize them toward the M1 phenotype, or treated them with anti-inflammatory cytokines (IL-4 and IL-13) to obtain the M2 phenotype. Morphological changes were observed in the microglia 24 h after the administration of inflammatory substances: the soma was enlarged, the number of processes was reduced, some cells lost their dendrites and swelled to an ovular shape, and ionized calcium-binding adapter molecule 1 (Iba1) was detected in the soma and its projections (Figure 1B–1F). These results support Tang and Le’s claim that both pro-inflammatory and anti-inflammatory factors can activate microglia [4].

**Figure 2:**
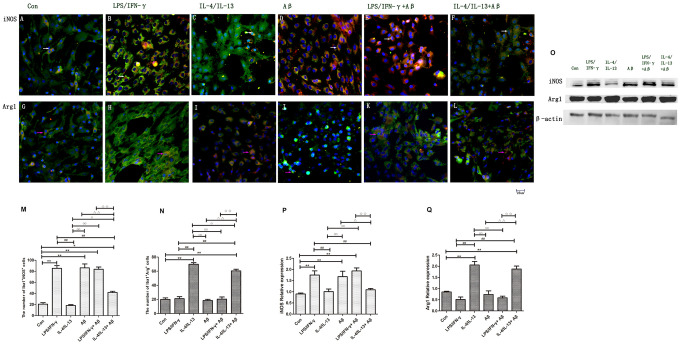
**Microglial polarization was induced by inflammatory factors and Aβ.** (**A**–**F**) Microglia in different groups were double-stained with anti-iNOS and anti-Iba1 antibodies and observed on a fluorescence microscope. Green represents Iba1^+^ cells, red represents iNOS^+^ cells and blue represents DAPI staining. The white arrows denote iNOS^+^Iba1^+^ cells. (**G**–**L**) Microglia in different groups were double-stained with anti-Arg1 and anti-Iba1 antibodies. Green represents Iba1^+^ cells, red represents Arg1^+^ cells and blue represents DAPI staining. White arrows denote Arg1^+^Iba1^+^ cells. Scale bars, 25 μm. (**M**, **N**) Quantitative data on the mean number of iNOS^+^Iba1^+^ or Arg1^+^Iba1^+^ cells (n=5). (**O**) Microglia in different groups were subjected to Western blotting to detect iNOS and Arg1. β-Actin was used as the internal control. (**P**, **Q**) Quantitative data on the relative protein levels of iNOS and Arg1 (n=3). Error bars, S.E.M. Compared with Con, *p<0.05, **p<0.01. Compared with LPS/IFN-γ, #*p*<0.05, ##*p*<0.01. Compared with IL-4/IL-13, ○*p*<0.05, ○○*p*<0.01. Compared with Aβ, Δ*p*<0.05, ΔΔ*p*<0.01. Compared with LPS/IFN-γ+Aβ, ↓*p*<0.05, ↓↓↓*p*<0.01. One-way ANOVA was performed with Tukey’s correction.

Polarized microglia are distinguishable by certain markers. Inducible nitric oxide synthase (iNOS), which metabolizes arginine into nitric oxide and citrulline, is a typical marker for M1 macrophages/microglia [15, 16], while Arg1, which metabolizes arginine into urea and ornithine, is often used as a marker of M2 macrophages/microglia [16]. We found that the number of iNOS^+^Iba1^+^ cells was considerably higher in the LPS/IFN-γ group than in the control group (*p<*0.01) (Figure 2A, 2B), whereas the number of Arg1^+^Iba1^+^ cells was greater in the IL-4/IL-13 group than in the control group (*p<*0.01) (Figure 2H, 2G). Western blotting confirmed that iNOS was significantly upregulated in the LPS/IFN-γ group (*p<*0.01), while Arg1 was upregulated in the IL-4/IL-13 group (*p<*0.01) (Figure 2O).

**Figure 3:**
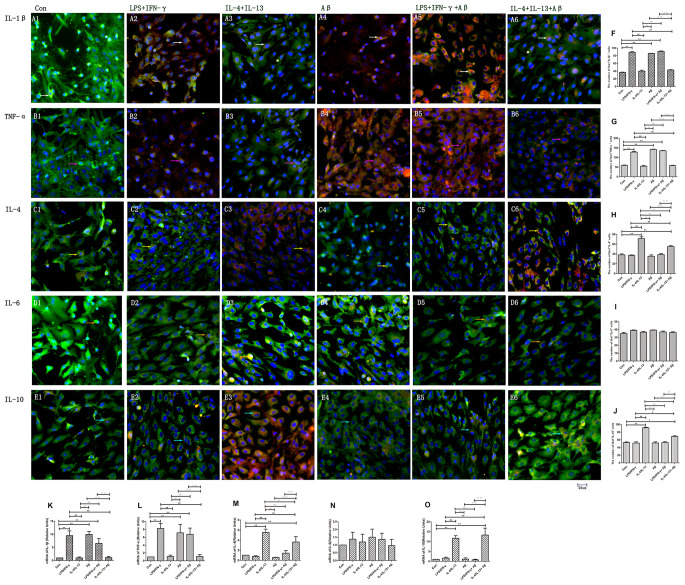
**Microglia produced various cytokines under different stimuli.** (**A**–**E**) Microglia in different groups were double-stained with antibodies against various cytokines and Iba1. (**A1**–**A6**) Double-staining for IL-1β and Iba1. (**B1**–**B6**) TNF-α and Iba1. (**C1**–**C6**) IL-4 and Iba1. (**D1**–**D6**) IL-6 and Iba1. (**E1**–**E6**) IL-10 and Iba1. Green represents Iba1^+^ cells, red represents cytokine-positive cells and blue represents DAPI staining. White arrows denote IL-1β^+^Iba1^+^ cells. Pink arrows denote TNF-α^+^Iba1^+^ cells. Yellow arrows denote IL-4^+^Iba1^+^ cells. Beige arrows denote IL-6^+^Iba1^+^ cells. Blue arrows denote IL-10^+^Iba1^+^ cells. (**F**–**J**) The numbers of double-positive cells in different groups (n=3). (**K**–**O**) Quantitative data on the relative mRNA levels of cytokines in microglia (n=3). Error bars, S.E.M. Compared with Con, *p<0.05, **p<0.01. Compared with LPS/IFN-γ, #*p*<0.05, ##*p*<0.01. Compared with IL-4/IL-13, ○*p*<0.05, ○○*p*<0.01. Compared with Aβ, Δ*p*<0.05, ΔΔ*p*<0.01. Compared with LPS/IFN-γ+Aβ, ↓*p*<0.05, ↓↓*p*<0.01. One-way ANOVA was performed with Tukey’s correction.

M1 and M2 macrophages/microglia can also be distinguished by the cytokines they release: M1 macrophages/microglia produce pro-inflammatory cytokines such as IL-1β and TNF-α, whereas M2 microglia secrete anti-inflammatory cytokines such as IL-4, IL-13 and IL-10 [4]. The numbers of IL-1β^+^ and TNF-α^+^ microglia were significantly greater in the LPS/IFN-γ group than in the control group (*p<*0.01) (Figure 3A1, A2, 3B1, B2), while there was no significant change in the number of IL-6^+^ cells (*p<*0.05) (Figure 3E1, E2). On the other hand, the numbers of IL-4^+^ and IL-10^+^ microglia were significantly greater in the IL-4/IL-13 group than in the control group (*p<*0.01) (Figure 3C1, C2, D1, D2). Consistently, *IL-1β* and *TNF-α* mRNA levels were upregulated in the LPS/IFN-γ group (*p<*0.01) (Figure 3K, 3L), while *IL-4* and *IL-10* mRNA levels were upregulated in the IL-4/IL-13 group (*p<*0.01) (Figure 3M, 3O).

### Media from polarized microglial cultures induced inflammatory factor production in astrocytes

**Figure 4:**
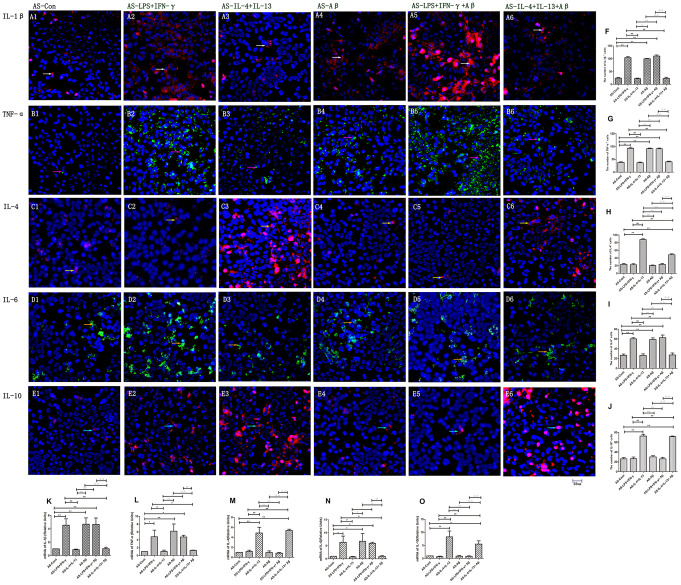
**Various cytokines were secreted by different astrocyte groups.** (**A**–**E**) Astrocytes in different groups were stained with antibodies against cytokines. (**A1**–**A6**) Staining for IL-1β (Red). (**B1**–**B6**) TNF-α (Green). (**C1**–**C6**) IL-4 (Red). (**D1**–**D6**) IL-6 (Green). (**E1**–**E6**) IL-10 (Red). Blue represents DAPI staining. White arrows denote IL-1β^+^ cells. Pink arrows denote TNF-α^+^ cells. Yellow arrows denote IL-4^+^ cells. Beige arrows denote IL-6^+^ cells. Blue arrows denote IL-10^+^ cells. (**F**–**J**) The numbers of positive cells in different groups (n=3). (**K**–**O**) Quantitative data on the relative cytokine levels in astrocytes (n=3). Error bars, S.E.M. Compared with AS-Con, *p<0.05, **p<0.01. Compared with AS-LPS/IFN-γ, #*p*<0.05, ##*p*<0.01. Compared with AS-IL-4/IL-13, ○*p*<0.05, ○○*p*<0.01. Compared with AS-Aβ, Δ*p*<0.05, ΔΔ*p*<0.01. Compared with AS-LPS/IFN-γ+Aβ, ↓*p*<0.05, ↓↓*p*<0.01. One-way ANOVA was performed with Tukey’s correction.

Astrocytes are involved in the inflammatory process in the CNS, and can produce both pro-inflammatory (IL-1β and TNF-α) and anti-inflammatory cytokines (IL-4 and IL-10) [17]. Thus, we analyzed whether polarized microglia of different phenotypes induced different inflammatory responses in astrocytes. Astrocytes (‘AS’) were treated with media from either LPS/IFN-γ-stimulated microglia (the AS-LPS/IFN-γ group) or IL-4/IL-13-stimulated microglia (the AS-IL-4/IL-13 group). The proportions of IL-1β^+^, TNF-α^+^ and IL-6^+^ astrocytes were all greater in the AS-LPS/IFN-γ group than in the AS-control group (*p<*0.01) (Figure 4A1, 4A2, 4B1, 4B2, 4D1, 4D2). On the other hand, the numbers of IL-4^+^ and IL-10^+^ astrocytes were greater in the AS-IL-4/IL-13 group than in the AS-control group (*p<*0.01) (Figure 4C1, 4C2, 4E1, 4E2). The mRNA levels of these factors in astrocytes followed similar trends (*p<*0.01) (Figure 4K–4O). Thus, LPS/IFN-γ-stimulated microglial medium upregulated pro-inflammatory factors in astrocytes, while IL-4/IL-13-stimulated microglial medium upregulated anti-inflammatory factors.

### Aβ induced M1 microglial polarization and pro-inflammatory factor secretion, but was selectively inhibited by IL-4/IL-13

Next, we exposed microglia to Aβ with or without various inflammatory factors to examine the activation mechanism of microglia under AD conditions. The following groups were assessed: control, Aβ, LPS/IFN-γ+Aβ, and IL-4/IL-13+Aβ. The number of iNOS^+^Iba1^+^ cells was considerably greater in the Aβ and LPS/IFN-γ+Aβ groups than in the control group (*p<*0.01) (Figure 2A, 2D, 2E), and iNOS protein expression was markedly elevated in these groups (*p<*0.01) (Figure 2O). In contrast, the number of iNOS^+^ microglia and the protein levels of iNOS were reduced in the IL-4/IL-13+Aβ group (*p<*0.01) (Figure 2A, 2F, 2O). Thus, anti-inflammatory factors prevented Aβ from inducing M1 microglial polarization. On the other hand, the number of Arg1^+^ microglia and the protein levels of Arg1 were significantly higher in the IL-4/IL-13+Aβ group than in the control group (*p<*0.01) (Figure 2G, 2L). The number of Arg1^+^ microglia was lower in IL-4/IL-13+Aβ group than in the IL-4/IL-13 group, although there was no significant difference in Arg1 protein expression between these groups (*p<*0.05) (Figure 2I, 2L). Thus, anti-inflammatory factors shifted Aβ-induced microglial polarization toward the M2 phenotype.

Aβ also induced the production of pro-inflammatory factors in microglia. The numbers of IL-1β^+^ and TNF-α^+^ microglia and the mRNA levels of these cytokines were significantly greater in the Aβ and LPS/IFN-γ+Aβ groups than in the control group (*p<*0.01) (Figure 3A5, 3B5), while they were markedly lower in the IL-4/IL-13+Aβ group than in the control group (*p<*0.01) (Figure 3C5, 3E5, 3K, 3L). Unexpectedly, The numbers of IL-1β^+^ and TNF-α^+^ microglia and the mRNA expressions did not differ significantly between the IL-4/IL-13 and IL-4/IL-13+Aβ groups (*p<*0.05) (Figure 3A5, 3A6, 3B5, 3B6, 3K, 3L). *IL-4* and *IL-10* levels were markedly greater in the IL-4/IL-13+Aβ group than in the control group (*p<*0.01) (Figure 3C6, 3E6). Although *IL-10* expression did not differ between the IL-4/IL-13 and IL-4/IL-13+Aβ groups (*p*>0.05), *IL-4* mRNA expression was lower in the IL-4/IL-13+Aβ group than in the IL-4/IL-13 group (*p<*0.05) (Figure 3M, 3O).

### Aβ-stimulated microglial medium induced pro-inflammatory factor expression in astrocytes, while Aβ coupled with IL-4/IL-13 improved their anti-inflammatory responses

We then treated astrocytes with media from microglia stimulated with Aβ. The following groups were assessed: AS-control, AS-Aβ, AS-LPS/IFN-γ+Aβ, and AS-IL-4/IL-13+Aβ. The numbers of IL-1β^+^, TNF-α^+^ and IL-6^+^ astrocytes and the mRNA levels of these pro-inflammatory factors were greater in the AS-Aβ and AS-LPS/IFN-γ+Aβ groups than in the AS-control group (*p<*0.01) (Figure 4A4, 4A5, 4B4, 4B5, 4D4, 4D5, 4K, 4L, 4N), but were lower in the AS-IL-4/IL-13+Aβ group than in the AS-control group (*p<*0.01) (Figure 4A6, 4B6, 4D6, 4K, 4L, 4N). Interestingly, these measures did not differ significantly between the AS-IL-4/IL-13 and AS-IL-4/IL-13+Aβ groups (p>0.05) (Figure 4A5, 4A6, 4B5, 4B6, 4D5, 4D6, 4K, 4L, 4N). On the other hand, the numbers of IL-4^+^ and IL-10^+^ cells and the mRNA levels of these anti-inflammatory factors were significantly greater in the AS-IL-4/IL-13+Aβ group than in the AS-control group (*p<*0.01) (Figure 4C6, 4E6, 4M, 4O).

### Pro-inflammatory factors and Aβ induced NF-κB expression, while anti-inflammatory factors enhanced Stat6 expression in microglia and microglial medium-induced astrocytes

**Figure 5:**
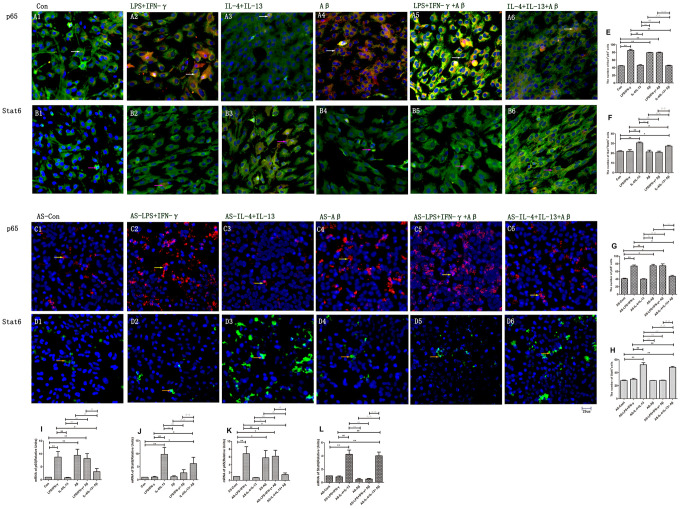
**Expression of p65 and Stat6 in different groups of microglia and astrocytes.** (**A**, **B**) p65 and Stat6 staining in microglia. (**A1**–**A6**) Double-staining for p65 and Iba1. (**B1**–**B6**) Stat6 and Iba1. Green represents Iba1^+^ cells, red represents p65^+^ or Stat6^+^ cells and blue represents DAPI staining. White arrows denote p65^+^Iba1^+^ cells. Pink arrows denote Stat6^+^Iba1^+^ cells. (**C**, **D**) p65 and Stat6 staining in astrocytes. (**C1**–**C6**) p65^+^ cells (Red and yellow arrows). (**D1**–**D6**) Stat6^+^ cells (Green and Beige arrows). (**E**–**H**) The numbers of positive cells in different groups (n=3). (**I**–**L**) Quantitative data on the relative levels of *p65* or *Stat6* (n=3). Error bars, S.E.M. Compared with Con or AS-Con, *p<0.05, **p<0.01. Compared with LPS/IFN-γ or AS-LPS/IFN-γ, #*p*<0.05, ##*p*<0.01. Compared with IL-4/IL-13 or AS-IL-4/IL-13, ○*p*<0.05, ○○*p*<0.01. Compared with Aβ or AS-Aβ, Δ*p*<0.05, ΔΔ*p*<0.01. Compared with LPS/IFN-γ+Aβ or AS-LPS/IFN-γ+Aβ, ↓*p*<0.05, ↓↓*p*<0.01. One-way ANOVA was performed with Tukey’s correction.

The NF-κB pathway component p65 is associated with inflammatory activity [6, 18]; thus, we measured p65 expression in microglia and microglial medium-treated astrocytes. The number of p65^+^ microglia and the mRNA levels of *p65* were greater in LPS/IFN-γ, Aβ and LPS/IFN-γ+Aβ microglial groups than in the control group (*p<*0.01) (Figure 5A1–5A3, 5I). Media originating from M1 microglia also activated the NF-κB pathway in astrocytes. The number of p65^+^ astrocytes and the mRNA levels of *p65* were greater in the AS-LPS/IFN-γ, AS-Aβ and AS-LPS/IFN-γ+Aβ groups than in the AS- control group (Figure 5C1–5C3, 5K). However, *p65* expression was downregulated in microglia treated with IL-4/IL-13+Aβ and in astrocytes treated with medium from these microglia (Figure 5A6, 5C6).

Given that Stat6 functions downstream of IL-4 [12], we also examined Stat6 expression in microglia and astrocytes. The number of Stat6^+^ cells and the mRNA levels of *Stat6* were greater in the IL-4/IL-13 microglial group than in the control group (*p<*0.01) (Figure 5B4, 5BJ). Interestingly, these measures were also upregulated in the IL-4/IL-13+Aβ microglial group (*p<*0.05) (Figure 5B6). In the astrocyte groups treated with microglial media, the number of Stat6^+^ cells and the mRNA levels of *Stat6* were greater in the AS-IL-4/IL-13 and AS-IL-4/IL-13+Aβ groups than in the AS-control group (*p<*0.01) (Figure 5D4, 5L). However, *Stat6* expression was lower in the AS-IL-4/IL-13+Aβ group than in the AS-IL-4/IL-13 group (*p<*0.01) (Figure 5D6, 5L).

## DISCUSSION

The crosstalk between astrocytes and microglia is crucial for many CNS functions, including development, neuronal development and immunological responses to diseases [19]. Proper bidirectional communication depends on a variety of molecules, including cytokines, neurotransmitters and metabolic substances. A review by Jha et al. indicated that pro-inflammatory substances (e.g., TNF-α, C1q) originating from microglia can influence astrocytes, for instance, by increasing TNF-α production and inducing astrocytosis. On the other hand, the astrocytic molecules LCN2 and ORM2 respectively enhance and inhibit microglial activity [3]. Thus, molecules produced by astrocytes can also alter microglial function.

It is well known that pro-inflammatory factors such as LPS and IFN-γ induce microglial polarization toward the M1 phenotype [20, 21], while anti-inflammatory factors such as IL-4 and IL-13 induce polarization toward the M2 phenotype [22]. However, it has been unclear how microglial polarization impacts astrocyte activity. Using different microglial media corresponding to these phenotypes, we found that M1 microglia stimulated pro-inflammatory astrocyte activity, while M2 microglia promoted anti-inflammatory astrocyte activity (Figure 5).

Recent studies have indicated that active astrocytes also exhibit different subtypes, of which the A1 subtype can be induced by classically activated microglia [23]. Another study demonstrated that the M1 phenotype marker iNOS stimulated pro-inflammatory processes by enhancing nitric oxide production in the CNS [24]. We found that pro-inflammatory molecules such as IL-1β and TNF-α were upregulated in M1 microglia; thus, these substances may have induced pro-inflammatory activity in astrocytes. A1 astrocyte-induced inflammation can subsequently trigger neuronal and glial apoptosis [23]. Moreover, these astrocytes are able to diffuse through the blood-brain barrier, damaging it in the process. Then, as an immune response, leukocytes and macrophages move toward the brain and begin to accumulate in the CNS [25], causing a detrimental cycle. Thus, astrocyte polarization induced by microglial polarization could further damage the CNS.

**Figure 6:**
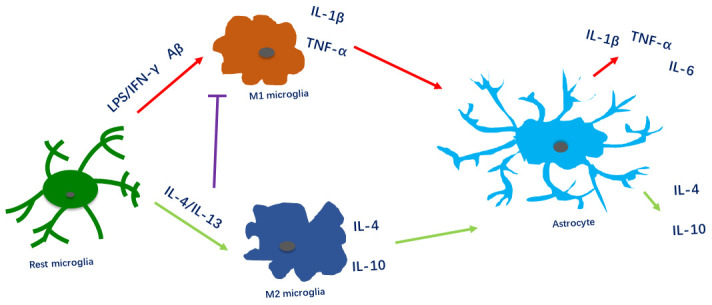
**Microglial polarization alters the inflammatory activity of astrocytes.** Pro-inflammatory factors and Aβ induce microglia to polarize toward the M1 phenotype and to produce IL-1β and TNF-α, thus stimulating pro-inflammatory activity in astrocytes. Anti-inflammatory factors (IL-4/IL-13) induce microglia to polarize toward the M2 phenotype and to produce IL-4 and IL-10, thus stimulating anti-inflammatory activity in astrocytes. Furthermore, anti-inflammatory factors reduce pro-inflammatory activity in microglia and astrocytes.

In contrast to M1 microglia, M2 microglia mainly secrete anti-inflammatory factors such as IL-10 and IL-4. A previous study demonstrated that IL-10 triggered TGF-β secretion from astrocytes, ultimately attenuating IL-1β production [26]. We observed that both M2 microglia and M2 microglial medium-induced astrocytes expressed anti-inflammatory factors (IL-4 and IL-10) with neuroprotective effects in the CNS (Figure 6).

The communication between different cells in the CNS is involved in the pathogenesis of AD. This is especially evident in the crosstalk between microglia and astrocytes. Astrocytic C3 activated through the microglial C3/C3a pathway increases Aβ pathology [8]. Aβ specifically activates neuroinflammation during the pathogenesis of AD by binding to certain receptors (CD14 and TLR4), thus stimulating pro-inflammatory activity in microglia [27, 28]. Here, we observed that M1 microglia were induced by Aβ alone and Aβ combined with LPS/IFN-γ. However, the induction of the M1 phenotype was not greater in the LPS/IFN-γ+Aβ group than in the Aβ group. In a previous study, Aβ plaques surrounded by M2 microglia were observed in 6-month-old PS1^M146L^/APP^751SL^ mice, but more abundant plaques were observed in 18-month-old mice when M2 microglia began to convert to the M1 phenotype [29]. Thus, the limited activation of M1 microglia in our study may have been due to regulatory mechanisms that maintain equilibrium between M2 and M1 activity, since we observed a small amount of M2 microglial polarization after administering Aβ and LPS/IFN-γ.

We also observed that IL-4/IL-13 shifted Aβ-induced microglial polarization toward the M2 phenotype. By inducing the production of M2 microglia, IL-4 and IL-13 may stimulate Aβ degradation. Indeed, in previous studies, M2 microglia reduced the deposition of Aβ (Aβ38, 40 and 42) both *in vitro* and *in vivo* [30, 31]. In the present study, the inflammatory crosstalk between astrocytes and microglia was also altered by Aβ treatment and microglial polarization. Aβ-induced M1 microglia enhanced the pro-inflammatory activity of astrocytes, whereas IL-4/IL-13 inhibited it. Thus, anti-inflammatory factors improved the protective crosstalk between astrocytes and microglia in AD.

The NF-κB pathway is recognized as one of the most important mechanisms involved in M1 microglial polarization. *In vitro*, LPS induces M1 polarization by activating the TLR4/TLR2/NF-κB pathway, which upregulates iNOS and major histocompatibility complex II [15]. We found that p65 levels increased in LPS/IFN-γ- and Aβ-treated microglia, as well as in astrocytes treated with media from these microglia. Thus, both LPS/IFN-γ and Aβ induced M1 microglial polarization and pro-inflammatory molecule secretion by stimulating the NF-κB pathway. A significant inflammatory trigger of the NF-κB pathway is the binding of molecules to TLR4 [32]. Aβ can serve as an agonist of CD14, which can then bind to TLR4 and activate the NF-κB pathway [33]. Our finding that p65 expression also increased in astrocytes suggested that M1 microglia activate pro-inflammatory astrocytes in an NF-κB-dependent manner. On the other hand, during M2 microglial polarization, we observed the upregulation of Stat6, which is well known to participate in the primary pathways of IL-4 and IL-13 [34]. A previous report indicated that Arg1 reduced inflammatory signaling in microglia by activating Stat6 pathways [12]. Our results indicated that the anti-inflammatory properties of M2 microglia and astrocytes were associated with Stat6.

In summary, this study demonstrated that M1/M2 microglial polarization alters the crosstalk between astrocytes and microglia. Moreover, Aβ promotes microglial polarization toward the M1 phenotype.

## MATERIALS AND METHODS

### Primary culture

Primary glial cells were obtained from the brain cortexes of one- to three-day-old newborn Sprague Dawley rats. The cortexes were removed aseptically from the whole brain, and the meninges were stripped carefully under a dissecting microscope. Then, the cortexes were cut into 1-mm x 1-mm pieces and digested with pancreatic enzymes for 10 min. The resulting cell suspension was cultured in Dulbecco’s modified Eagle’s medium (DMEM) supplemented with 10% fetal bovine serum in a 15-mL poly-L-lysine-coated flask and incubated at 37°C and 5% CO_2_. After two weeks, the mixed glial cells were shaken at 120 rpm on a gyratory shaker for 4 h. For microglia, the floating cells were collected and reseeded in DMEM/F12 supplemented with 10% fetal bovine serum in 2-mL poly-L-lysine-coated plates. Microglial growth reached the logarithmic phase after four weeks. For astrocytes, the adherent cultures were cultivated in DMEM supplemented with 10% fetal bovine serum. Astrocyte growth reached the logarithmic phase after three weeks.

### Treatment of microglia with different stimulants

To observe microglial polarization, we used typical stimuli: LPS and IFN-γ for M1 polarization, and anti-inflammatory cytokines (IL-4 and IL-13) for M2 polarization [4, 16]. The microglia were divided into six groups: the control group (Con), the LPS/IFN-γ treatment group (LPS/IFN-γ), the IL-4/IL-13 treatment group (IL-4/IL-13), the Aβ1-24 treatment group (Aβ), the LPS/IFN-γ and Aβ1-24 co-treatment group (LPS/IFN-γ+Aβ) and the IL-4/IL-13 and Aβ1-24 co-treatment group (IL-4/IL-13+Aβ). Microglia in the LPS/IFN-γ and LPS/IFN-γ+Aβ groups were incubated with LPS (100 ng/mL, Sigma) and IFN-γ (100 U/mL, Santa Cruz Biotechnology) for 24 h. For the IL-4/IL-13 and IL-4/IL-13+Aβ groups, the microglia were incubated with IL-4 (20 ng/mL, Santa Cruz Biotechnology) and IL-13 (20 ng/mL, Santa Cruz Biotechnology) for 24 h. For the LPS/IFN-γ+Aβ and IL-4/IL-13+Aβ groups, the microglia were pre-incubated with Aβ1-24 (1 ng/mL, Sigma) for 2 h before LPS/IFN-γ or IL-4/IL-13 were added, and then the microglia were incubated with both Aβ and these inflammatory substances for 24 h.

### Co-culture of astrocytes with microglia-conditioned medium

To determine whether microglia of different phenotypes induced astrocytes, we cultured primary astrocytes in conditioned media from different microglia [23]. Primary astrocytes were divided into six groups: the astrocyte control group (AS-Con), astrocytes treated with LPS/IFN-γ-induced microglial medium (AS-LPS/IFN-γ), astrocytes treated with IL-4/IL-13-induced microglial medium (AS-IL-4/IL-13), astrocytes treated with Aβ1-24-induced microglial medium (AS-Aβ), astrocytes treated with LPS/IFN-γ- and Aβ1-24-induced microglial medium (AS-LPS/IFN-γ+Aβ) and astrocytes treated with IL-4/IL-13- and Aβ1-24-induced microglial medium (AS-IL-4/IL-13+Aβ). Media from the different microglial groups were collected and centrifuged (400 x *g*, 7 min). The supernatants were then collected and incubated with the astrocytes for 24 h.

### Immunohistochemistry

Cells were fixed with 4% paraformaldehyde for 10 min and then washed in phosphate-buffered saline (PBS). After being blocked with 5% bovine serum albumin containing 0.5% Triton X-100 for 1 h at 37°C, the microglia were incubated with the following primary antibodies at 4°C overnight: mouse anti-Iba1 (1:200, Bioss, Beijing, China), rabbit anti-iNOS, anti-Arg1, anti-IL-1β, anti-TNF-α, anti-IL-4, anti-IL-6 and anti-IL-10 (1:200, Bioss). After being washed in PBS three times, the cells were incubated with the following secondary antibodies at 37°C for 1 h: Alexa Fluor 488-conjugated goat anti-rabbit IgG (1:200, Bioss) or Alexa Fluor Cy3-conjugated goat anti-mouse IgG (1:200, Bioss). After being washed in PBS three times, the cells were incubated with 4′,6-diamidino-2-phenylindole (DAPI; Biyuntian, China) for 5 min. The cells were then washed in PBS and observed under a 7266-fluorescence microscope (Leica, Japan).

### Quantitative real-time PCR

Total RNA was extracted from logarithmic-phase microglia using Trizol (Abcam), and cDNA was obtained using a cDNA Synthesis Kit. Quantitative real-time PCR was performed on a PCR cycler (Bio-Rad CFX96) with synthetic primers and SYBR Green (Sangon Biotech, China). Samples were subjected to the following reaction conditions: 95°C for 3 min, followed by 45 cycles of 95°C for 10 s, renaturation for 30 s and 60°C for 30 s. The 2^-ΔΔCt^ method was used to calculate relative mRNA levels. The sequences of the primers were as follows: *IL-1β* forward, 5’-GAGCTGAAAGCTCTCCACCT-3’ and reverse, 5’-TTCCATCTTCTTCTTTGGGT-3’; *TNF-α* forward, 5’-GCCCACGTCGTAGCAA-3’ and reverse, 5’-GTCTTTGAGATCCATGCCAT-3’; *IL-6* forward, 5’-AGAAGACCAGAGCAGATTTT-3’ and reverse, 5’- GAGAAAAGAGTTGTGCAATG-3’; *IL-4* forward, 5’- CTTTGAACCAGGTCACAG-3’ and reverse, 5’-CTCGTTCTCCGTGGTGTT-3’; *IL-10* forward, 5’-CAGAAATCAAGGAGCATTTG-3’ and reverse, 5’- CTGCTCCACTGCCTTGCTTT-3’; *p65* forward, 5’- CTGTTTCCCCTCATCTTTCCCT-3’ and reverse, 5’- CTGGTCCTGTGTAGCCATTGA-3’; *Stat6* forward, 5’- ATGCTTCCATGCAACTCAGC-3’ and reverse, 5’-GCTCCTGAAAAGATGGCAGT -3’.

### Western blotting

For protein extraction, 1 mL of NP-40 lysis buffer (Biyuntian) was transferred to each well of a six-well plate of microglia on ice for 30 min, and the cells were then centrifuged at 12,000 rpm for 10 min. The soluble protein solutions were then mixed with 5x sample buffer (Biyuntian) and boiled at 90^o^C for 10 min. Equal amounts of protein (60 μg) from each sample were separated via 10% sodium dodecyl sulfate polyacrylamide gel electrophoresis (Sigma) and transferred to polyvinylidene fluoride membranes (Millipore, Billerica, MA, USA). The membranes were blocked in 5% milk with Tris-buffered saline for 1 h, and then were incubated with the following primary antibodies at 4°C overnight: rabbit anti-iNOS, anti-Arg1 (1:500, Bioss) and rabbit anti-actin (1:1000, Santa Cruz Biotechnology). The membranes were washed three times in Tris-buffered saline with Tween and then incubated with the secondary antibody (peroxidase-conjugated IgG, 1:5000, Santa) at room temperature for 2 h. Binding antibodies were visualized using enhanced chemiluminescence on an imaging system (Amersham Imager 600).

### Statistical analysis

All experiments were performed at least three times independently. Data were analyzed with one-way analysis of variance (ANOVA) followed by Bonferroni’s multiple comparison test (SPSS 20.0, IBM, USA). The results are expressed as the mean ± standard error of the mean (S.E.M.).

## References

[1] Reemst K, Noctor SC, Lucassen PJ, Hol EM. The indispensable roles of microglia and astrocytes during brain development. Front Hum Neurosci. 2016; 10:566. 10.3389/fnhum.2016.00566 27877121 PMC5099170

[2] Pascual O, Ben Achour S, Rostaing P, Triller A, Bessis A. Microglia activation triggers astrocyte-mediated modulation of excitatory neurotransmission. Proc Natl Acad Sci USA. 2012; 109:E197–205. 10.1073/pnas.1111098109 22167804 PMC3268269

[3] Jha MK, Jo M, Kim JH, Suk K. Microglia-astrocyte crosstalk: an intimate molecular conversation. Neuroscientist. 2019; 25:227–40. 10.1177/1073858418783959 29931997

[4] Tang Y, Le W. Differential roles of M1 and M2 microglia in neurodegenerative diseases. Mol Neurobiol. 2016; 53:1181–94. 10.1007/s12035-014-9070-5 25598354

[5] Shi Q, Chowdhury S, Ma R, Le KX, Hong S, Caldarone BJ, Stevens B, Lemere CA. Complement C3 deficiency protects against neurodegeneration in aged plaque-rich APP/PS1 mice. Sci Transl Med. 2017; 9:eaaf6295. 10.1126/scitranslmed.aaf6295 28566429 PMC6936623

[6] Shi X, Cai X, Di W, Li J, Xu X, Zhang A, Qi W, Zhou Z, Fang Y. MFG-E8 selectively inhibited Aβ-induced microglial M1 polarization via NF-κB and PI3K-Akt pathways. Mol Neurobiol. 2017; 54:7777–88. 10.1007/s12035-016-0255-y 27844286

[7] Xie L, Wu Q, Tang Y, Zhuang Z, Zhao N, Huang B, Yu S. [Study on electroacupuncture promoting the polarization of M2 phenotype microglia cells in hippocampus of AD model rats]. Zhonghua Zhongyiyao Zazhi. 2018; 33:1816–20. https://kns.cnki.net/kcms/detail/detail.aspx?FileName=BXYY201805028&DbName=CJFQ2018

[8] Lian H, Litvinchuk A, Chiang AC, Aithmitti N, Jankowsky JL, Zheng H. Astrocyte-microglia cross talk through complement activation modulates amyloid pathology in mouse models of Alzheimer’s disease. J Neurosci. 2016; 36:577–89. 10.1523/JNEUROSCI.2117-15.2016 26758846 PMC4710776

[9] Litvinchuk A, Wan YW, Swartzlander DB, Chen F, Cole A, Propson NE, Wang Q, Zhang B, Liu Z, Zheng H. Complement C3aR inactivation attenuates tau pathology and reverses an immune network deregulated in tauopathy models and Alzheimer’s disease. Neuron. 2018; 100:1337–53.e5. 10.1016/j.neuron.2018.10.031 30415998 PMC6309202

[10] Qin C, Liu Q, Hu ZW, Zhou LQ, Shang K, Bosco DB, Wu LJ, Tian DS, Wang W. Microglial TLR4-dependent autophagy induces ischemic white matter damage via STAT1/6 pathway. Theranostics. 2018; 8:5434–51. 10.7150/thno.27882 30555556 PMC6276098

[11] Shi H, Wang XL, Quan HF, Yan L, Pei XY, Wang R, Peng XD. Effects of betaine on LPS-stimulated activation of microglial M1/M2 phenotypes by suppressing TLR4/NF-κB pathways in N9 cells. Molecules. 2019; 24:367. 10.3390/molecules24020367 30669620 PMC6359206

[12] Cai W, Dai X, Chen J, Zhao J, Xu M, Zhang L, Yang B, Zhang W, Rocha M, Nakao T, Kofler J, Shi Y, Stetler RA, et al. STAT6/Arg1 promotes microglia/macrophage efferocytosis and inflammation resolution in stroke mice. JCI Insight. 2019; 4:e131355. 10.1172/jci.insight.131355 31619589 PMC6824303

[13] Chen KY, Wang LC. Stimulation of IL-1β and IL-6 through NF-κB and sonic hedgehog-dependent pathways in mouse astrocytes by excretory/secretory products of fifth-stage larval angiostrongylus cantonensis. Parasit Vectors. 2017; 10:445. 10.1186/s13071-017-2385-0 28950910 PMC5615811

[14] Neal M, Luo J, Harischandra DS, Gordon R, Sarkar S, Jin H, Anantharam V, Désaubry L, Kanthasamy A, Kanthasamy A. Prokineticin-2 promotes chemotaxis and alternative A2 reactivity of astrocytes. Glia. 2018; 66:2137–57. 10.1002/glia.23467 30277602 PMC6240381

[15] Cunha C, Gomes C, Vaz AR, Brites D. Exploring new inflammatory biomarkers and pathways during LPS-induced M1 polarization. Mediators Inflamm. 2016; 2016:6986175. 10.1155/2016/6986175 28096568 PMC5209629

[16] Orihuela R, McPherson CA, Harry GJ. Microglial M1/M2 polarization and metabolic states. Br J Pharmacol. 2016; 173:649–65. 10.1111/bph.13139 25800044 PMC4742299

[17] Fakhoury M. Microglia and astrocytes in Alzheimer’s disease: implications for therapy. Curr Neuropharmacol. 2018; 16:508–18. 10.2174/1570159X15666170720095240 28730967 PMC5997862

[18] Shah A, Silverstein PS, Singh DP, Kumar A. Involvement of metabotropic glutamate receptor 5, AKT/PI3K signaling and NF-κB pathway in methamphetamine-mediated increase in IL-6 and IL-8 expression in astrocytes. J Neuroinflammation. 2012; 9:52. 10.1186/1742-2094-9-52 22420994 PMC3338363

[19] Fang M, Yuan Y, Rangarajan P, Lu J, Wu Y, Wang H, Wu C, Ling EA. Scutellarin regulates microglia-mediated TNC1 astrocytic reaction and astrogliosis in cerebral ischemia in the adult rats. BMC Neurosci. 2015; 16:84. 10.1186/s12868-015-0219-6 26608466 PMC4660684

[20] Yao A, Liu F, Chen K, Tang L, Liu L, Zhang K, Yu C, Bian G, Guo H, Zheng J, Cheng P, Ju G, Wang J. Programmed death 1 deficiency induces the polarization of macrophages/microglia to the M1 phenotype after spinal cord injury in mice. Neurotherapeutics. 2014; 11:636–50. 10.1007/s13311-013-0254-x 24853068 PMC4121443

[21] Jang E, Lee S, Kim JH, Kim JH, Seo JW, Lee WH, Mori K, Nakao K, Suk K. Secreted protein lipocalin-2 promotes microglial M1 polarization. FASEB J. 2013; 27:1176–90. 10.1096/fj.12-222257 23207546

[22] Francos-Quijorna I, Amo-Aparicio J, Martinez-Muriana A, López-Vales R. IL-4 drives microglia and macrophages toward a phenotype conducive for tissue repair and functional recovery after spinal cord injury. Glia. 2016; 64:2079–92. 10.1002/glia.23041 27470986

[23] Liddelow SA, Guttenplan KA, Clarke LE, Bennett FC, Bohlen CJ, Schirmer L, Bennett ML, Münch AE, Chung WS, Peterson TC, Wilton DK, Frouin A, Napier BA, et al. Neurotoxic reactive astrocytes are induced by activated microglia. Nature. 2017; 541:481–87. 10.1038/nature21029 28099414 PMC5404890

[24] Tapias V, Hu X, Luk KC, Sanders LH, Lee VM, Greenamyre JT. Synthetic alpha-synuclein fibrils cause mitochondrial impairment and selective dopamine neurodegeneration in part via iNOS-mediated nitric oxide production. Cell Mol Life Sci. 2017; 74:2851–74. 10.1007/s00018-017-2541-x 28534083 PMC5524146

[25] Sofroniew MV. Astrocyte barriers to neurotoxic inflammation. Nat Rev Neurosci. 2015; 16:249–63. 10.1038/nrn3898 25891508 PMC5253239

[26] Norden DM, Fenn AM, Dugan A, Godbout JP. TGFβ produced by IL-10 redirected astrocytes attenuates microglial activation. Glia. 2014; 62:881–95. 10.1002/glia.22647 24616125 PMC4061706

[27] Origlia N, Criscuolo C, Arancio O, Yan SS, Domenici L. RAGE inhibition in microglia prevents ischemia-dependent synaptic dysfunction in an amyloid-enriched environment. J Neurosci. 2014; 34:8749–60. 10.1523/JNEUROSCI.0141-14.2014 24966375 PMC4069353

[28] Liu S, Liu Y, Hao W, Wolf L, Kiliaan AJ, Penke B, Rübe CE, Walter J, Heneka MT, Hartmann T, Menger MD, Fassbender K. TLR2 is a primary receptor for Alzheimer’s amyloid β peptide to trigger neuroinflammatory activation. J Immunol. 2012; 188:1098–107. 10.4049/jimmunol.1101121 22198949

[29] Jimenez S, Baglietto-Vargas D, Caballero C, Moreno-Gonzalez I, Torres M, Sanchez-Varo R, Ruano D, Vizuete M, Gutierrez A, Vitorica J. Inflammatory response in the hippocampus of PS1M146L/APP751SL mouse model of Alzheimer’s disease: age-dependent switch in the microglial phenotype from alternative to classic. J Neurosci. 2008; 28:11650–61. 10.1523/JNEUROSCI.3024-08.2008 18987201 PMC6671312

[30] Cherry JD, Olschowka JA, O’Banion MK. Arginase 1+ microglia reduce Aβ plaque deposition during IL-1β-dependent neuroinflammation. J Neuroinflammation. 2015; 12:203. 10.1186/s12974-015-0411-8 26538310 PMC4634600

[31] Latta CH, Sudduth TL, Weekman EM, Brothers HM, Abner EL, Popa GJ, Mendenhall MD, Gonzalez-Oregon F, Braun K, Wilcock DM. Determining the role of IL-4 induced neuroinflammation in microglial activity and amyloid-β using BV2 microglial cells and APP/PS1 transgenic mice. J Neuroinflammation. 2015; 12:41. 10.1186/s12974-015-0243-6 25885682 PMC4350455

[32] Mussbacher M, Salzmann M, Brostjan C, Hoesel B, Schoergenhofer C, Datler H, Hohensinner P, Basílio J, Petzelbauer P, Assinger A, Schmid JA. Cell type-specific roles of NF-κB linking inflammation and thrombosis. Front Immunol. 2019; 10:85. 10.3389/fimmu.2019.00085 30778349 PMC6369217

[33] Fassbender K, Walter S, Kühl S, Landmann R, Ishii K, Bertsch T, Stalder AK, Muehlhauser F, Liu Y, Ulmer AJ, Rivest S, Lentschat A, Gulbins E, et al. The LPS receptor (CD14) links innate immunity with Alzheimer's disease. FASEB J. 2004; 18:203–5. 10.1096/fj.03-0364fje 14597556

[34] McCormick SM, Heller NM. Commentary: IL-4 and IL-13 receptors and signaling. Cytokine. 2015; 75:38–50. 10.1016/j.cyto.2015.05.023 26187331 PMC4546937

